# The enemy’s gaze: Immersive virtual environments enhance peace promoting attitudes and emotions in violent intergroup conflicts

**DOI:** 10.1371/journal.pone.0222342

**Published:** 2019-09-11

**Authors:** Yossi Hasson, Noa Schori-Eyal, Daniel Landau, Béatrice S. Hasler, Jonathan Levy, Doron Friedman, Eran Halperin

**Affiliations:** 1 Psychology Department, The Hebrew University, Jerusalem, Israel; 2 School of Psychology, The Interdisciplinary Center, Herzliya, Israel; 3 School of Communications, The Interdisciplinary Center, Herzliya, Israel; 4 Department of Media, School of Arts, Design and Architecture, Aalto University, Espoo, Finland; University of Milan, ITALY

## Abstract

Perspective-taking is essential for improving intergroup relations. However, it is difficult to implement, especially in violent conflicts. Given that immersive virtual reality (VR) can simulate various points of view (POV), we examined whether it can lead to beneficial outcomes by promoting outgroup perspective-taking, even in armed conflicts. In two studies, Jewish-Israelis watched a 360° VR scene depicting an Israeli-Palestinian confrontation from different POVs–outgroup’s, ingroup’s while imagining outgroup perspective or ingroup’s without imagined perspective-taking. Participants immersed in the outgroup’s POV, but not those who imagined the outgroup’s perspective, perceived the Palestinians more positively than those immersed in the ingroup’s POV. Moreover, participants in the outgroup’s POV perceived the Palestinian population in general more favorably and judged a real-life ingroup transgression more strictly than those in the ingroup’s POV, even five months after VR intervention. Results suggest that VR can promote conflict resolution by enabling effective perspective-taking.

## Introduction

Intergroup conflicts are one of the major problems facing human society. From the beginning of 21^st^ century, armed intergroup conflicts cost the lives of more than 1 million people worldwide [1], and during 2017 nearly 70 million men, women and children were uprooted from their homes as a result of wars, violence and persecution [2]. Many violent conflicts are rooted in political, racial, and religious dissensions [3], and are difficult to overcome because of multiple psychological barriers [4, 5]. Perspective-taking–imagining the world (or specific situations) from another’s point of view or imagining oneself in another’s shoes–can potentially help overcome such barriers, and was found to reduce negative outgroup evaluation [6], outgroup mistrust [7], intergroup biases [8], and to increase prosocial emotions and behavior towards the outgroup [9].

Although perspective-taking is beneficial in promoting intergroup reconciliation [10], it is rarely implemented effectively in conflictual contexts for several reasons. First, individuals naturally embrace their ingroup’s perspective rather than the outgroup’s [11] and they are often unable (i.e., lack of skills) to adopt a reliable perspective of the outgroup. Instead, the perspective taken is superficial and lacks vivid or detailed views of the other, which can generate misunderstanding [12]. Second, individuals are unwilling (i.e., lack of motivation) to perceive distress and suffering of the enemy group members [13, 14] because it might evoke guilt over harmful actions or wrongdoing of the ingroup [15]. In addition, taking the perspective of the enemy group may cost individuals credibility with their ingroup and even lead to social exclusion [12], and the benefits of perspective-taking are limited by reactive egoism, in which perspective takers react defensively as they imagine the target to be biased by self-interest [16]. Such difficulty in accurately taking the outgroup’s perspective in conflictual contexts can sometimes backfire, leading to even more negative consequences including less tolerance and helping behavior [17–19].

How, then, can perspective-taking be made more practicable and effective in intergroup conflicts? As traditional perspective-taking is highly arduous in prolonged and violent conflicts, and insufficient to substantially change hostile attitudes and emotions, a different and innovative approach was explored. We examined whether immersion in the enemy’s point of view through virtual reality (VR) would enable effective perspective-taking that leads to more positive emotions and attitudes towards the adversary, and consequently reduces intergroup tensions.

VR has an almost limitless potential to simulate reality from different perspectives and overcome technical and fundamental challenges that could not be met in the real world. Consequently, recent years have seen a rise in systematic examination of how innovative VR technology could be harnessed to promote perspective-taking and thus to improve interpersonal and intergroup relations. Previous VR-based studies on perspective-taking used various approaches, including virtual embodiment [20], engaging with a virtual environment from another person’s point of view [21] or simulating the perceptual experience of another [22]. Their findings indicated that VR can facilitate perspective-taking by immersing people in another person’s character or position, and through it reduce negative attitudes, increase empathy, and promote helping behavior. However, these studies demonstrated the beneficial effects of VR-based perspective-taking in relatively non-competitive and controlled settings related to contexts that posed little threat. Moreover, very few studies showed any impact beyond the VR context and over a long period of time.

The present research aimed to examine the effectiveness of perspective-taking through VR in the challenging context of prolonged, violent intergroup conflicts where it is most needed, but also most challenging to implement. Specifically, we tested the effectiveness of immersive perspective-taking relative to traditional perspective-taking or control in the context of the intractable Israeli-Palestinian conflict and examined its immediate effects and long-term impact beyond the VR context. In two studies we tested the hypotheses that immersive exposure to the rival outgroup’s point of view (POV) would: (*i*) lead to more positive emotions and attitudes towards the outgroup, compared with the ingroup’s POV (Studies 1&2); (*ii*) be more effective than traditional perspective-taking instructions (Study 1); and (*iii*) lead to positive outcomes that are long-lasting and generalize beyond the immediate VR context (i.e., other conflict situations and outgroup members; Study 2).

To this end, we created a 360° VR scene presenting a confrontation between Israeli soldiers and a Palestinian couple at a military checkpoint. The scripted scene reflects the realities of the ongoing Israeli-Palestinian conflict, and the friction and tensions that culminate in military checkpoints in which soldiers and civilians come into precarious contact. The same scene was filmed from two POVs—the Palestinian couple’s POV and the Israeli soldiers’ POV. In both studies Jewish-Israeli participants were either immersed in the Palestinian/outgroup POV (i.e., immersive perspective-taking) or in the Israeli/ingroup POV that reflects the ingroup perspective people naturally take in real life (i.e., control)^1^. Study 1 included a comparison between the two POVs and a traditional perspective-taking condition in which participants were immersed in the Israeli/ingroup POV while instructed to implement traditional outgroup perspective-taking instructions. In Study 2 we focused on the comparison between the outgroup’s POV and the ingroup’s POV, and also measured the effect of the manipulation five months later beyond the VR context in a real-life event.

## Study 1

The goal of Study 1 was to test the effect of immersive perspective-taking and traditional perspective-taking on positive emotions and attitudes compared with a control condition. We hypothesized that immersive perspective-taking would induce more positive emotions, positive outgroup appraisals, attribution of benign intentions, and support for compensation compared with a control condition. We further hypothesized that these effects would be stronger in comparison to the difference between traditional perspective-taking and the control condition.

## Materials and methods

The study procedures were approved by the Institutional Review Board at the Interdisciplinary Center Herzliya, and all participants completed an online consent form. To minimize risk, participants who did not feel well, suffered from epileptic episodes, or used psychopharmacological drugs were disqualified for participations because VR environments may lead to dizziness and nausea. Participants with high scores on posttraumatic stress disorder or depression scales were also disqualified to avoid exposure to the conflict-related emotionally intense VR clip.

### Participants

One hundred and twelve Jewish-Israeli students (*M*_age_ = 23.92, *SD*_age_ = 4.26; 86 women) from an Israeli university participated in the study in exchange for course credit. A power analysis based on a recent study that used 360° VR and examined empathy in an intergroup context ([23]; *d* = .5) indicated that a sample size of 32 would be sufficient to detect an effect at 80% power. Indeed, similar sample sizes used in the domain of immersive technologies were sufficiently powered to detect differences between experimental conditions (see [24] for a meta-analysis). However, because our study was conducted with individuals involved in an intractable conflict that is characterized by low levels of empathy but high levels of fear, we expected the effect size to be smaller. Therefore, we decided to substantially increase the sample size.

### Procedure

**Fig 1:**
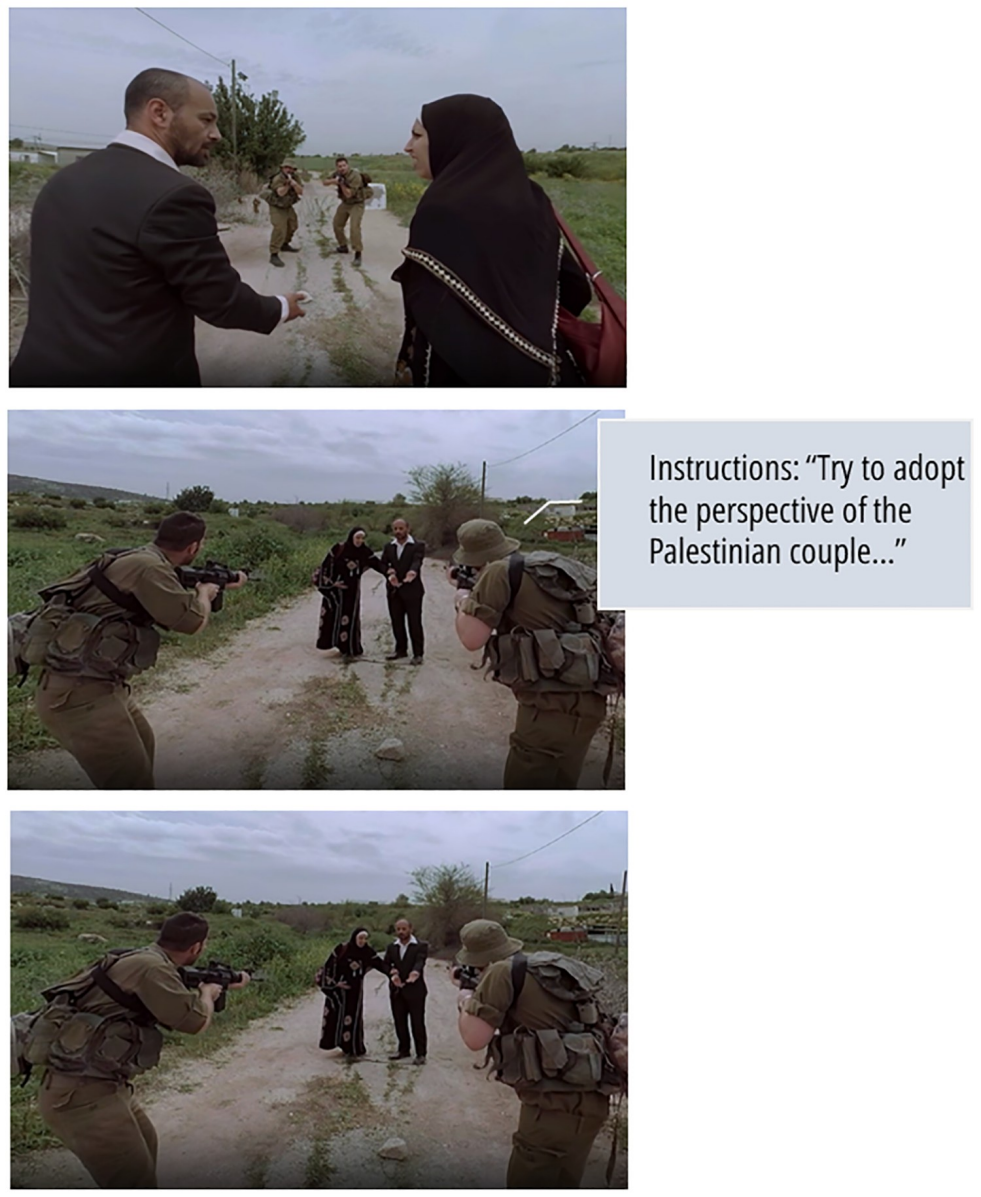
Participants’ point of view in each condition. Top: Palestinian/outgroup POV (i.e., immersive perspective-taking). Middle: Israeli/ingroup POV + imagined outgroup perspective-taking instructions (i.e., traditional perspective-taking). Bottom: Israeli/ingroup POV (i.e., control).

Participants read a brief description of a confrontation between Israeli soldiers and a Palestinian couple at a military checkpoint. They were then randomly assigned to watch a 1-minute 360° VR scene^2^ depicting the interaction they had previously read about in one of three conditions: (*i*) Palestinian/outgroup POV (i.e., immersive perspective-taking; *n* = 37); (*ii*) Israeli/ingroup POV + imagined outgroup perspective-taking instructions (i.e., traditional perspective-taking; *n* = 38); and (*iii*) Israeli/ingroup POV with no such instructions (i.e., control; *n* = 37). Participants in the immersive perspective-taking and the control conditions were merely instructed to pay attention and listen carefully while watching the scene. Participants in the traditional perspective-taking condition were also instructed to adopt the perspective of the Palestinian couple by imagining that they are one of the Palestinians, experiencing the situation through his/her eyes and being in his/her shoes. These instructions were adapted from previous studies on traditional perspective-taking [12]. The three POVs are illustrated in Fig 1.

In the scene, a Palestinian man and an apparently pregnant Palestinian woman approach two Israeli soldiers at a military roadblock. The soldiers instruct the Palestinians to halt and begin inspecting them, and an altercation unfolds. The scene ends when the Palestinian man reaches into his jacket, and in response the soldiers point their rifles at the couple (see S1 Appendix for media materials). Participants watched the scene wearing a VR headset (Samsung Gear VR), which provided interactive panoramic 360° head-tracking VR with corresponding directed sound to experience the filmed interaction. Immediately after watching the scene participants completed a questionnaire regarding the interaction that included measures of their emotions (i.e., empathic emotions and fear) towards the Palestinians in the scene; appraisals of the Palestinian couple’s attitudes and behaviors as perceived in the VR scene; future benign, non-violent, intentions of the Palestinian man after the ambiguous end of the VR scene; and support for compensation for the Palestinian couple, if they were misidentified as militants and mistakenly shot and wounded by the soldiers. In addition, participants completed a demographic questionnaire that included their gender, age, and political orientation.

### Measures

#### Empathic emotions and fear

Following Porat et al. [25] we used three items to assess the degree to which participants experienced emotions toward the outgroup members (ranging from 1—Not at all to 7—Very much): empathic emotions (empathy, sympathy, r = .75), and fear of the Palestinian couple (e.g., “Following the scene, to what extent do you currently feel each of the feelings below: empathy, sympathy, fear”).

#### Positive appraisals

Four items were developed for this study to assess the degree to which the Palestinian couple was perceived positively (e.g., "The Palestinians felt threatened"; ranging from 1 –not at all, to 7 –very much; α = .69).

#### Attribution of future benign intentions

As the VR scene ended ambiguously before the Palestinian man took action, one item, developed for this study, assessed the likelihood that the Palestinian man intended to do as he stated when reaching into his jacket: "The Palestinian intended to issue documents" (ranging from 1 –*zero-probability* to 7—*Absolute certainty*).

#### Support for compensation

Two items, developed for this study, assessed the degree to which participants supported compensation for the Palestinian couple in case they were misidentified as militants and mistakenly shot and wounded by the soldiers: "I would support providing financial compensation to the Palestinians for harming them"; "I would support an official Israeli apology to the Palestinians for harming them" (ranging from 1—strongly disagree to 7—strongly agree; r = .68).

#### Political ideology

Following Porat et al. [25] we assessed political ideology regarding foreign policy and security issues by using a single item, ranging from 1—e*xtreme right*, to 7—e*xtreme left*).

## Results

To examine the experimental effects on the dependent variables, we ran a series of univariate ANOVAs with the condition (immersive perspective-taking, traditional perspective-taking, or control) as a between-subject variable. Fisher’s LSD post hoc tests were used to examine differences between the three conditions. To eliminate potential effect of participants’ demographic variables, we controlled for gender, age, and political ideology in which some were found to be correlated with our outcome variables (see S1 Table). The pattern of results remained similar when not controlling for these demographic variables.

### Empathic emotions

The results revealed a significant effect of condition on empathy towards the Palestinians in the scene [*F*(2, 109) = 8.41, *p* < .001, *d* = .80] indicating that participants in the immersive perspective-taking and the traditional perspective-taking conditions displayed significantly greater empathy towards the Palestinians than participants in the control condition (see Table 1 for means and SDs).

**Table 1:** Descriptive statistics of the outcome variables by condition (Study 1).

Outcome	Condition		
	Control	Traditional Perspective-taking	Immersive Perspective-taking
Empathic emotions	3.84 (1.34)	4.93 (1.54)	4.76 (1.32)
Fear	3.41 (1.96)	2.82 (1.69)	2.49 (1.63)
Positive appraisals	4.94 (1.06)	5.22 (1.03)	5.66 (.89)
Attribution of future benign intentions	4.86 (.82)	5.13 (1.34)	5.62 (.83)
Support for compensation	4.34 (1.60)	4.71 (1.84)	5.31 (1.60)

### Fear

There was no significant effect of condition on fear of the Palestinians in the scene [*F*(2, 109) = 1.53, *p* = .22, *d* = .34]. A pairwise comparison between the immersive perspective-taking and the control conditions was also insignificant (*p* = .108) though approaching significance. The difference between the traditional perspective-taking and the control condition was not significant (*p* = .174).

### Positive appraisals

We found that the experimental conditions had a significant effect on positive appraisals of the Palestinian couple [*F*(2, 109) = 3.93, *p =* .023, *d* = .54]. Participants in the immersive perspective-taking condition had significantly higher scores than those participants in the control condition (*p* = .006), whereas participants in the traditional perspective-taking condition did not significantly differ from those in the control condition (*p* = .15).

### Attribution of future benign intentions

We also found a significant effects on attributing future benign intentions of the Palestinian couple [*F*(2, 109) = 4.17, *p =* .018, *d* = .56]. Participants in the immersive perspective-taking condition had significantly higher scores than those participants in the control condition (*p* = .005), whereas participants in the traditional perspective-taking condition did not significantly differ from those in the control condition (*p* = .28).

### Support for compensation

In addition, the conditions significantly differed regarding ratings of supported compensation for the Palestinians if wrongfully wounded [*F*(2, 109) = 3.12, *p =* .048, *d* = .49]. Participants in the immersive perspective-taking condition had significantly higher scores than those in the control condition (*p* = .014), whereas participants in the traditional perspective-taking condition did not significantly differ from those in the control condition (*p* = .293).

The post hoc analyses indicated that participants in the immersive perspective-taking condition had significantly higher scores than those in the control condition, whereas participants in the traditional perspective-taking condition did not significantly differ from those in the control condition (except for empathic emotions). However, no significant differences were found between the immersive perspective-taking condition and the traditional perspective-taking condition (*p* = .074–1).

Study 1 indicated that while both kinds of perspective-taking increased empathy towards the Palestinian couple, immersive perspective-taking produced much more meaningful effects on all other pro-reconciliation variables, including positive appraisals, attribution of future intentions and support for compensation. We conducted the next study with the goal of examining the effect of immersive perspective-taking on more general perceptions of the outgroup beyond the specific VR context as well as additional behavioral-related variables. In addition, we tested whether the general perceptions of the outgroup are mediated by any of the effects found in the specific VR context. We also aimed to examine potential long-term effects of the 1-minute VR intervention in a real-life context. Having established that compared with the control condition, immersive perspective-taking was superior to the traditional perspective-taking in most of the outcome variables, we focused on the comparison between immersive perspective-taking and the control condition.

## Study 2

Study 2 was conducted at two time-points with 5 months difference to examine whether immersive perspective-taking has an immediate and a long-lasting effect on general perceptions of the outgroup and in a real-life intergroup event. Having found in Study 1 that VR-based perspective-taking was more effective than traditional perspective-taking instructions, we focused in Study 2 on the comparison between immersive perspective-taking and control conditions. We hypothesized that immersive perspective-taking would lead to more positive response toward the outgroup in the VR context and consequently increase positive perceptions of the outgroup in general and in a real-life event beyond the VR context.

## Materials and methods

### Participants

One hundred^3^ Jewish-Israeli students (*M*_age_ = 23.85, *SD*_age_ = 2.44; 71 women) participated in the study in exchange for course credit. The sample size was calculated using a power analysis based on the effect on empathy found in Study 1 (*d* = .80) indicated that a sample size of 52 would be sufficient to detect an effect at 80% power. Because we considered a dropout in the second time-point measure we doubled the initial sample size. Five months after VR intervention, we contacted the participants and invited them to take part in a new study (without mentioning the first session and without providing information that could connect the two parts of the study). Fifty-five participants responded to the invitation and took part in the second measurement (55% of the baseline sample, *M*_age_ = 23.86, *SD*_age_ = 2.88, 40 women). Previous research indicates that dropout ratios of 30% to 70% are at most weakly associated with bias [26]. Nevertheless, to examine whether our results were skewed due to participant dropout between the two measurements as a result of their gender, age, political ideology, or the condition to which they were assigned, we conducted a logistic regression. None of these variables were found to be a significant predictor of dropout (all p’s > .41) and accounted for less than 1.5% of the variance of attrition, indicating that the dropout was mostly random.

### Procedure

In the first session Jewish-Israeli student participants were randomly assigned to watch the same 360° VR scene presented in Study 1 from either the Palestinian/outgroup POV (*n* = 50) or the Israeli/ingroup POV^4^ (*n* = 50). Participants were not given specific instructions in either condition. Immediately after watching the scene, participants completed a questionnaire that included measures of emotions (i.e., empathic emotions and fear) toward the Palestinians in the scene. In addition, they indicated their general perceptions of the Palestinian population including dehumanization and perceived threat. These variables correspond with the core appraisals of empathy [27] and fear [28] respectively, that were measured regarding the specific Palestinian couple who was presented in the VR clip. Finally, participants provided demographic information as in Study 1.

Five months after watching the scene, we contacted the participants and invited them to take part in a new study without mentioning the first session and without providing information that could connect the two parts of the study. Fifty-five participants (Palestinian/outgroup POV: *n* = 26; Israeli/ingroup POV: *n* = 29) responded to the invitation and took part in the second measurement. Participants completed the same measures of general perceptions of Palestinians—dehumanization and perceived threat. To tap into behavioral tendencies, we presented participants with a series of short vignettes describing ambiguous situations in which a Palestinian could be perceived as an attacker or not. In each vignette, participants had to indicate whether or not they would shoot the Palestinian ("Shoot/No-shoot Dilemma" measure). This task simulates the decision faced by the soldiers in the clip: whether a suspect poses real threat and should be shot (which is the point in which the scene ended).

Finally, participants were asked questions about a real-life conflict-related event that was receiving intense media coverage and sparked a widespread public debate at that time—the results of a military trial against an Israeli soldier who had shot and killed an incapacitated Palestinian militant that had previously stabbed other Israeli soldiers in Hebron, in March 2016 [29]. While shooting an incapacitated enemy combatant (*hors de combat*) is a violation of the International Humanitarian Law and of the Israel Defense Forces rules of engagement, the soldier’s actions and their consequences sparked a heated and widespread public debate. After reading a short informative description of the Hebron incident participants completed measures of moral emotions towards the soldier’s actions, perceived morality of his actions, and severity of appropriate punishment.

### Measures

#### VR context—Toward the Palestinians in the scene

**Empathic emotions and fear**. Same as in Study 1 (*empathy*, *sympathy*, *r* = .68).

#### General context—Toward the Palestinian population

**Dehumanization**. Adapted from McDonald et al. [30] we assessed dehumanization of the Palestinians in general by using the following item: "Psychological research shows that people tend to attribute different levels of humanity to different groups. The following scale represents the Palestinians’ level of humanity, with 0 indicating very little humanity and 100 indicating high humanity. Please choose the number that represents the degree to which you see Palestinians as humans". The scale ranged from 0—*not at all human*; to 100—*very much human*. Item was reverse scored.

**Perceived threat**. Two items adapted from Canetti-Nisim et al. [31] to assess the degree to which participants viewed Palestinians as a threat to Israel’s security: "The Palestinians are a danger to the very existence of Israel"; "The Palestinians jeopardize Israel’s Jewish character" (ranging from 1—*strongly disagree* to 7—*strongly agree*; *r* = .62).

**Shoot/No-shoot Dilemma**. Eight short vignettes describing ambiguous situations in which a Palestinian could be perceived as an attacker of not. Each vignette was followed by the question: “should the person described in the scenario be shot with the intent to wound him/her?” the responses were yes/no. Based on participants’ responses to the vignettes we calculated a bias measure (c), based on the standard signal detection formula [32], which averages the z-score corresponding to the hit rate and the false alarm rate, with a loglinear correction^5^ for extreme values [32,33]. Positive responses were coded as false alarms for all scenarios (i.e., they were treated as noise trials), except for the most extreme scenario where there was considerable evidence that the target was indeed the attacker. For this scenario, a positive response was coded as a hit (i.e., this was treated as a signal trial). This resulted in a measure of bias, to which we refer as the "shoot bias": whether the targets should be shot with intent to wound them. Positive scores indicate a cautious or conservative bias, a score of 0 indicates a neutral bias, whereas negative scores indicate an incautious or liberal bias.

#### Real-life context

**Moral emotions**. Three items were used to measure participants’ level of moral emotions over the soldier’s actions in Hebron (i.e., *guilt*, *shame*, and *pride* [reversed]; ranging from 1 –*not at all*, to 7 –*very much*; *α* = .86).

**Morality judgment of the soldier’s actions** in Hebron was measured using a single item ("to what degree to you think the soldier’s action was moral?"; ranging from 1 –*very immoral*, to 7 –*very moral*).

**Severity of punishment for the soldier** was measured using a single item ("What punishment or reward should the soldier receive for his actions?"; ranging from 1 –*release from prison and receive honorable mention*, to 7 –*receive a long prison term*).

**Political ideology**. Same as in Study 1.

## Results

To examine the effects of the two conditions on the dependent variables, we ran a series of univariate ANOVAs with condition (immersive perspective-taking vs. control) as a between-subject variable while controlling for the same demographic variables as in Study 1 (see S2 Table for correlations of all variables).

### Time 1 (immediately after VR intervention)

We replicated the significant effect of condition on empathy [*F*(1, 98) = 7.96, *p* = .006, *d* = .58], and also found an effect on fear [*F*(1, 98) = 6.39, *p* = .013, *d* = .52] towards the Palestinians in the scene (see Table 2 for means and SDs). In addition, participants in the immersive perspective-taking condition were less dehumanizing of the Palestinian population [*F*(1, 98) = 7.96, *p* = .006, *d* = .58] and perceived them as less threatening [*F*(1, 98) = 4.27, *p* = .042, *d* = .42] compared with participants in the control condition.

**Table 2:** Descriptive statistics of the outcome variables by condition (Study 2).

Outcome		Condition	
		Control	Immersive Perspective-taking
T1 (upon VR intervention)			
VR context–toward the Palestinians in the scene			
	Empathic emotions	3.74 (1.47)	4.43 (1.52)
	Fear	3.44 (1.86)	2.72 (1.53)
General context–toward the Palestinian population			
	Dehumanization	33.4 (27.30)	22.20 (23.76)
	Perceived threat	4.73 (1.65)	4.26 (1.44)
T2 (five months later)			
General context–toward the Palestinian population			
	Dehumanization	41.38 (27.22)	31.15 (24.71)
	Perceived threat	4.81 (1.44)	4.04 (1.26)
	Shoot/No-shoot Dilemma	-.14 (.51)	.15 (.49)
Real-life context			
	Moral emotions	3.99 (1.30)	4.76 (1.20)
	Moral judgment of the soldier action	3.24 (1.68)	2.50 (1.14)
	Severity of punishment for the soldier	5.21 (1.29)	5.81 (1.02)

### Time 2 (five months later)

Even Five months after the first phase, and although the actual manipulation lasted for about one minute only, we found a persistent effect on the general perception of the outgroup such that participants in the immersive perspective-taking condition were still less dehumanizing of the Palestinian population [*F*(1, 53) = 5.70, *p* = .021, *d* = .67] and perceived them as less threatening [*F*(1, 53) = 6.02, *p* = .018, *d* = .69] compared with participants in the control condition. We also found that participants in the immersive perspective-taking condition were more cautious and made fewer "shoot" decisions when confronted with ambiguous vignettes [*F*(1, 53) = 4.16, *p* = .047, *d* = .58] than participants in the control condition.

In response to questions about the real-life event of the soldier’s shooting of an incapacitated Palestinian militant, participants in the immersive perspective-taking condition experienced higher levels of moral emotions [*F*(1, 53) = 8.28, *p* = .006, *d* = .81]; judged the soldier’s actions more harshly [*F*(1, 53) = 5.17, *p* = .027, *d* = .64]; and supported a more severe punishment for the shooting, [*F*(1, 53) = 6.17, *p* = .016, *d* = .70], compared with the control condition.

**Fig 2:**
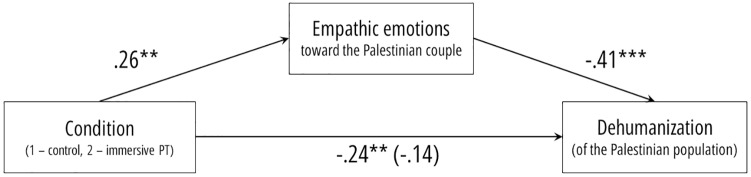
Empathic emotions toward the Palestinian couple presented in the VR scene mediate the effect of manipulated VR POV on dehumanization of the Palestinian population in general.

To examine whether the responses toward the Palestinian couple in the VR scene were generalized toward the Palestinian population in general, we conducted two mediation analyses employing the procedure of Hayes [34] PROCESS bootstrapping macro (model 4; 5,000 iterations). Each analysis included an emotional response toward the Palestinian couple (i.e., empathic emotions or fear) and a general perception of the Palestinian population that corresponds with the core appraisals of the emotional response (i.e., dehumanization or perceived threat respectively). The first model was specified with condition as the independent variable, empathic emotions as the mediator variable, and dehumanization as the outcome variable (Fig 2). As expected, the total effect (*b* = -.25, 95% CI = [-.42, -.07], *t* = -2.82, *p* = .006) was reduced when empathic emotions were added as a mediator (*b* = -.14, 95% CI = [-.31, .23], *t* = -1.7, *p* = .092). The indirect effect through the mediator was statistically different from zero (*b* = .11, 95% CI = [-.21, -.03]). Participants in the immersive perspective-taking felt more empathic emotions toward the Palestinian couple, which in turn, was associated with less dehumanization of Palestinian in general.

**Fig 3:**
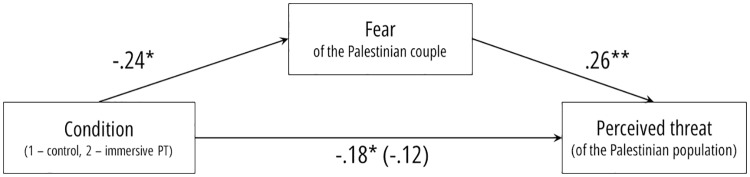
Fear of the Palestinian couple presented in the VR scene mediate the effect of manipulated VR POV on perceived threat of the Palestinian population in general.

The second model was specified with condition as the independent variable, fear as the mediator variable, and perceived threat as the outcome variable (Fig 3). As expected, the total effect (*b* = -.18, 95% CI = [-.35, -.01], *t* = -2.06, *p* = .042) was reduced when fear was added as a mediator (*b* = -.12, 95% CI = [-.29, .05], *t* = -1.4, *p* = .178). The indirect effect through the mediator was statistically different from zero (*b* = -.06, 95% CI = [-.15, -.01]). Participants in the immersive perspective-taking condition felt less fear of the Palestinian couple, which in turn, was associated with less perceived threat of Palestinians in general.

Finally, we meta-analyzed Studies 1&2 using fixed effects in which the mean effect size was weighted by sample size and comparing the control condition with the immersive perspective-taking condition. Under these aggregated conditions, participants in the outgroup POV felt significantly greater empathic emotions (M d = .61, Z = 3.94, p < .001, 95% confidence interval (CI) = [.31, .92] and less fear (M d = .61, Z = 2.88, p = .002, 95% CI = [.14, .73]) compared with those in the ingroup POV.

## Discussion

The goal of the current project was to examine, in the violent and prevalent context of intergroup conflicts, whether immersive VR can surpass traditional perspective-taking in producing beneficial outcomes. Our findings suggest that virtually taking a rival outgroup’s perspective via immersion in the outgroup POV has greater positive impact both immediately after VR intervention and several months later, compared with immersion in the ingroup POV with or without traditional perspective-taking instructions. While both forms of perspective-taking were effective in increasing empathy, only immersive perspective-taking reduced fear of the outgroup, induced more positive outgroup attributions, and led to stricter moral judgment of ingroup transgressors.

The research findings indicate a mechanism in which the response to the specific virtual context expanded to the outgroup in general. Immersive exposure to the outgroup’s POV led to increased empathy and decreased fear toward the virtual outgroup members that consequently were generalized as greater humanization and less perceived threat of the rival outgroup in general. More importantly, the effect of the 1-minute VR intervention was detected five months after immersion in the outgroup’s POV, increasing moral emotions and stricter judgement of a real-life ingroup transgression. We believe that experiencing the outgroup perspective in a virtual environment left a great impact on the participants, enabling a change in the real-world with less effort or motivation that are needed in the traditional perspective-taking [16]. To our knowledge this is the first study showing a long-term impact of a VR perspective-taking intervention in conflictual context, by merely altering the camera perspective in a 360° video.

The significant advances and lower cost of VR devices and production of VR stimuli are making the technology increasingly accessible to the mass public via a variety of smartphone devices and appropriate headsets that enhance the immersive experience [35]. This is particularly true for 360° VR videos, which are a simpler technique compared to using a first-person embodiment illusion. Hence, our findings can lead to developing applicable VR-based interventions as a tool for enabling perspective-taking in conflict on a global scale.

Together with the potential theoretical and applicable contributions, several limitations of the present project bear mentioning along with associated future research directions. First, the samples were biased in terms of gender, as most participants were women. Given that women are more associated with empathic-related traits and behaviors than men do [36] it might be easier for them to take the perspective of the other group. Although we statistically controlled for the participants’ gender to avoid bias in our findings, future studies should use samples with equal number of men and women.

Second, we used the ingroup perspective as the control condition because in intergroup context people tend to favor their ingroup and adopt its perspective rather than the outgroup [6]. We believe that not only did participants in our study favor the ingroup, as group members tend to do in intergroup contexts; the perspective of Israeli soldiers was the natural perspective for them to adopt. Military service is mandatory in Israel for both men and women, and all participants in the research were either soldiers themselves only a couple of years prior to participating in the study, or had close friends and relatives who served in the military. However, it is possible that not all participants identified with their ingroup perspective as we would expect. Future studies should overcome this concern by validating before the VR experience which perspective is in favor for each participant.

Beyond the potential limitations described above, several questions remain: Do all group members respond favorably, or would some react adversely when being exposed to the enemy’s POV, albeit virtually? Under what conditions might exposure to the rival outgroup’s POV create a backlash effect and increase negative perceptions of the rival outgroup? Would this intervention work in other socio-political contexts? Is immersive perspective-taking beneficial only for members of the dominant group, or would it have salutary effects on the low-power group as well? Would being first-person embodied in the outgroup perspective lead to stronger effect than the change in POV used in the present research, or would it create resistance and backfire? Future studies that replicate our findings and address these questions would lend additional support to our assertion that VR technology is a powerful tool for perspective-taking interventions in intergroup conflicts, with the potential of causing significant changes using increasingly available and compelling technologies.

## Supporting information

S1 Appendix Immersive 360° VR stimuli. (DOCX)

S1 Footnotes (DOCX)

S1 Table Bivariate correlations for all variables of Study 1. (DOCX)

S2 Table Bivariate correlations for all variables of Study 2. (DOCX)

S1 Dataset Dataset for Study 1. Contains all the raw data used for analysis in Study 1.
(XLSX)

S2 Dataset Dataset for Study 2. Contains all the raw data used for analysis in Study 2.
(XLSX)
