# Supplementary material for: The enemy’s gaze: Immersive virtual environments enhance peace promoting attitudes and emotions in violent intergroup conflicts
Source: PLoS One. 2019 Sep 11;14(9):e0222342. doi: 10.1371/journal.pone.0222342 (PMC6738917; doi:10.1371/journal.pone.0222342)
Supplement: S1 Appendix — (DOCX) [file pone.0222342.s001.docx]

**S1 Appendix - Immersive 360**° **VR stimuli**

The mechanism of the immersive 360° VR stimuli is based on the theory of Active Vision and Sensorimotor Contingencies (SCs) that emphasize the role of motor actions on perception. During the studies, participants wore a head-mounted display (HMD) with head-tracking, so the visual input of the virtual environment was synchronized with their head movement, simulating the way they perceive the real world. Such implementation of the SCs, in which participants used their body to perceive the stimuli, generated a strong sense of presence (i.e., ‘feeling of being there’). To increase the sense of presence, we used a 360° VR that may generate a more realistic environment compared to other VR technologies that use synthetic computer graphics.

Below are the links to the VR stimuli:

- Outgroup POV (immersive perspective-taking): <https://youtu.be/khfQG_7vFic>
- Ingroup POV (control): <https://youtu.be/bE2P-5trNqo>

Note: When watching the videos on a device with no head-tracking (e.g., a desktop computer) the experience is qualitatively different and may result in lower psychological impact.
