# Supplementary material for: The enemy’s gaze: Immersive virtual environments enhance peace promoting attitudes and emotions in violent intergroup conflicts
Source: PLoS One. 2019 Sep 11;14(9):e0222342. doi: 10.1371/journal.pone.0222342 (PMC6738917; doi:10.1371/journal.pone.0222342)
Supplement: S1 Footnotes — (DOCX) [file pone.0222342.s002.docx]

**S1 Footnotes.**

^1^ In intergroup context, people tent to adopt their ingroup perspective rather than the outgroup perspective. The Israeli soldiers in the VR scene are part of the Israeli Jewish population in which all the participants belong to. In addition, because the military service in Israel is mandatory for both men and women, all the participants served as soldiers prior to their academic degree or have relatives and friends who were in the army. Thus, it is likely that the soldiers’ perspective is adopted by Israeli Jews even without intervention and best represents a control condition, relatively to the other perspectives.

^2^ Prior to the scripted scene there was a 30-second "orientation" phase in which participants looked around the immersed environment.

^3^ Five participants were removed from the analyses due to technical issues during the experiment. However, we obtained similar results when including them in the analyses.

^4^ The scene was also filmed from a bystander perspective that was pilot-tested for the purposes of another study and is not included in the report.

^5^ The loglinear correction is a common approach for correcting extreme values in signal detection measurements. It involves adding .5 to the number of hits and false alarms and 1 to the number of signal trials and the number of noise trials. It is needed because if the hit or false alarm rate is 0 or 1, mathematically the bias score cannot be determined because 0 and 1 correspond to z scores of negative infinity or infinity. Because shooting with the intent to kill is an extreme and serious action there were many participants who did not choose this option for any scenario, resulting in a hit rate of 0. Therefore, some correction for extreme values was necessary. We chose the loglinear approach because it is a common and effective way of dealing with extreme values [1] and it can be applied even in the absence of extreme values [2]. Therefore, we applied it to all bias scores.

**References**

1. Hautus MJ (1995) Corrections for extreme proportions and their biasing effects on estimated values of d′. *Behav Res Methods Instrum Comput* 27(1):46-51.
2. Stanislaw H, Todorov N (1999) Calculation of signal detection theory measures. *Behav Res Methods Instrum Comput* 31(1):137-149.
