# Supplementary material for: The enemy’s gaze: Immersive virtual environments enhance peace promoting attitudes and emotions in violent intergroup conflicts
Source: PLoS One. 2019 Sep 11;14(9):e0222342. doi: 10.1371/journal.pone.0222342 (PMC6738917; doi:10.1371/journal.pone.0222342)
Supplement: S1 Table — (DOCX) [file pone.0222342.s003.docx]

**S1 Table.** Bivariate Correlations for All Variables of Study 1.

|  | 1 | 2 | 3 | 4 | 5 | 6 | 7 | 8 |
| --- | --- | --- | --- | --- | --- | --- | --- | --- |
| 1. Empathic emotions | - |  |  |  |  |  |  |  |
| 2. Fear | -.318^**^ | - |  |  |  |  |  |  |
| 3. Positive appraisals | .557^***^ | -.341^***^ | - |  |  |  |  |  |
| 4. Attribution of future benign intentions | .392^***^ | -.198^*^ | .466^***^ | - |  |  |  |  |
| 5. Support for compensation | .633^***^ | -.218^*^ | .440^***^ | .352*^**^ | - |  |  |  |
| 6. Gender | .063 | .361^***^ | -.079 | .020 | .055 | - |  |  |
| 7. Age | .162 | -.135 | .026 | .113 | .160 | -.100 | - |  |
| 8. Political ideology | -.469^***^ | .414^***^ | -.467^***^ | -.389*^**^ | -.379^***^ | .142 | -.269^**^ | - |

**p* < .05. ***p* < .01 *** *p* < .001.
