# Supplementary material for: The enemy’s gaze: Immersive virtual environments enhance peace promoting attitudes and emotions in violent intergroup conflicts
Source: PLoS One. 2019 Sep 11;14(9):e0222342. doi: 10.1371/journal.pone.0222342 (PMC6738917; doi:10.1371/journal.pone.0222342)
Supplement: S2 Table — (DOCX) [file pone.0222342.s004.docx]

**S2 Table.** Bivariate Correlations for All Variables of Study 2.

|  | 1 | 2 | 3 | 4 | 5 | 6 | 7 | 8 | 9 | 10 | 11 | 12 |
| --- | --- | --- | --- | --- | --- | --- | --- | --- | --- | --- | --- | --- |
| 1. Empathic emotions |  |  |  |  |  |  |  |  |  |  |  |  |
| 1. Fear | -.373^***^ |  |  |  |  |  |  |  |  |  |  |  |
| 1. Dehumanization (T1) | -.511^***^ | .388^***^ |  |  |  |  |  |  |  |  |  |  |
| 1. Perceived threat (T1) | -.254^*^ | .400^***^ | .460^***^ |  |  |  |  |  |  |  |  |  |
| 1. Dehumanization (T2) | -.397^**^ | .370^**^ | .635^***^ | .412^**^ |  |  |  |  |  |  |  |  |
| 1. Perceived threat (T2) | -.307^*^ | .474^***^ | .282^*^ | .590^***^ | .511^***^ |  |  |  |  |  |  |  |
| 1. Moral emotions | .395^***^ | -.146 | -.308^*^ | -.476^***^ | -.367^**^ | -.301^*^ |  |  |  |  |  |  |
| 1. Moral judgment of the soldier action | -.460^**^ | .194 | .357^**^ | .494^***^ | .406^**^ | .343^*^ | -.559^***^ |  |  |  |  |  |
| 1. Severity of punishment for the soldier | -.397^**^ | .252 | .268^*^ | .542^***^ | .303^*^ | .545^***^ | -.503^***^ | .551^***^ |  |  |  |  |
| 1. Shoot/No-shoot Dilemma | .319^*^ | -.242 | -.253 | -.382^**^ | -.271^*^ | -.425^***^ | .138 | -.069 | -.324^*^ |  |  |  |
| 1. Gender | -.051 | .221^*^ | .387^***^ | .239^*^ | .420^***^ | .093 | -.223 | .176 | .194 | .056 |  |  |
| 1. Age | .178 | -.264^**^ | -.277^**^ | -.155 | -.348^**^ | -.122 | .083 | -.185 | -.127 | -.070 | -.521^***^ |  |
| 1. Political ideology | .350^***^ | -.244^*^ | -.374^***^ | -.510^***^ | -.437^***^ | -.491^***^ | .506^***^ | -.407^**^ | -.567^***^ | .073 | -.254^*^ | .192 |

**p* < .05. ***p* < .01 *** *p* < .001.
